# Supplementary material for: Regulating Citywide Inclusive Sanitation (CWIS) in Colombia
Source: Int J Environ Res Public Health. 2022 May 6;19(9):5669. doi: 10.3390/ijerph19095669 (PMC9100875; doi:10.3390/ijerph19095669)
Supplement: Supplementary file 1 [file ijerph-19-05669-s001.zip › ijerph-1668960-SI.pdf]

Supplementary Material

Supplementary S1: Supplementary material for the methodology

Table S1. Questionnare for Key Stakeholders at a National Level

<table>
<tr><td colspan="3">Introduction: I am Analía Saker. As I mentioned in the email, this interview is to understand your thoughts about the urban sanitation policy in Colombia. The interview will take approximately 30 minutes. It is part of my dissertation and aims to understand the regulatory barriers to reach 100% urban sanitation coverage in Colombia. I will try to publish the results in a journal. Your contributions will be anonymised. In the reporting, we would only use either job titles or the institution typology (e.g. urban utility, NGO, Ministry of Housing, City and Territory, Developing Bank). You are free to withdraw from this research at any time.
Spanish:
Hola, mi nombre es Analía Saker. Como mencioné previamente por email, esta entrevista se realizará para entender los problemas relacionados a saneamiento básico en Tumaco y cómo encararlos. Esta entrevista se demorará aproximadamente 30 minutos y hace parte de un trabajo de investigación de la Universidad de Oxford en Inglaterra. Intentaremos publicar los resultados en una revista académica. Sus contribuciones serán anónimas y el reporte se utilizará únicamente el nombre del cargo o la institución para la que trabaja (por ejemplo: empresa prestadora de servicios públicos domiciliarios, ONG, Alcaldía de Tumaco). Usted es libre de retirarse de esta investigación en cualquier punto.
• Are you happy to continue with the interview? Make a note of the consent in your notes.
¿Está de acuerdo con continuar con la entrevista? Tomar nota de la respuesta
• I would like to record the interview to enable us to transcribe it. The recording will not be shared, and it will be deleted once transcription is completed and checked. Do you consent to us recording the interview? - Make a note of the consent in your notes and start recording if agreed.
Quisiera grabar esta entrevista para transcribirla después. La grabación no se compartirá y se borrará una vez la entrevista se haya transcrito. ¿Da su consentimiento para grabar esta entrevista? Tomar nota de la respuesta</td></tr>
<tr><td>Required information</td><td>Questions</td><td>Prompts to gather the required information</td></tr>
<tr><td rowspan="3">Current position details to evidence sampling frame. [it is important in these early questions to build rapport.</td><td>What is your current job?
¿Cuál es su trabajo actual?</td><td>What is the job title?
Cuál es el título de dicho trabajo
What is it that you do in that role? [conversation starter question – note details gathered below]
Cuáles son sus responsabilidades en ese rol?</td></tr>
<tr><td>Are you happy for us to use this job title for reporting purposes?
¿Está conforme con que utilice el título de su posición para reportar en la investigación?</td><td></td></tr>
<tr><td>What has been you experience in urban sanitation?
Desde su entidad, qué proyectos ha adelantado en saneamiento urbano?</td><td>Ask details about specific projects
Preguntar detalles sobre proyectos específicos</td></tr>
<tr><td>Perception of Colombian urban sanitation situation</td><td>What is your opinion about Colombia performance in urban sanitation coverage?
Cuál es su opinión sobre el</td><td>Do you think Colombia is performing better than its neighbouring countries?
¿En su opinión, Colombia tiene un mejor desempeño que los otros países de la región?</td></tr>
</table>

Applicability of CWIS to tackle the urban sanitation issues	Do you know what Citywide Inclusive Sanitation (CWIS) is?<br>¿Sabe lo que es CWIS?	*Ask follow-up questions:*<br>*Preguntas de seguimiento:*<br>*If yes, where did you hear about it? What do you know about it? How would you define CWIS?*<br>*Si sí, ¿En qué contexto ha escuchado/interactuado con CWIS? ¿Cómo lo definiría?*<br>*If no, explain them the concept*<br>*Si no, explicar el concepto.*
	Do you think that incorporating CWIS as an urban sanitation approach would help Colombia reach 100% urban sanitation coverage?<br>¿Piensa que CWIS puede servir como una nueva estrategia para alcanzar la cobertura nacional de saneamiento urbano?	*If yes, how so? If not, why not?*<br>*Si sí, ¿Cómo? Si no, ¿Por qué?*
Barriers to reach 100% urban sanitation coverage	What do you think the principal barriers are to achieve 100% urban sanitation coverage in Colombia?<br>¿Cuáles piensa que son las principales barreras para lograr el 100% de la cobertura en saneamiento urbano?	*Do you think it might be a lack of finance, lack of incentives, regulatory barriers, unprepared institutions?*<br>*¿Opina que puede ser una falta de financiación, falta de incentivos, barreras regulatorias o instituciones débiles?*
	Do you think the existing regulatory framework poses barriers to reach 100% urban sanitation?<br>*¿Piensa que el marco regulatorio actual supone barreras para alcanzar esta meta?*	*If so, how?*<br>*Si sí, ¿Cómo?*
	In your opinion, what specific regulations should be adapted to incorporate CWIS as a more holistic sanitation approach?<br>En su opinión, ¿Qué regulaciones específicas deberían adaptarse para incorporar CWIS como una estrategia más holística para lograr la meta de saneamiento urbano?	*Inquire abour specific regulations that are barriers or require further development for CWIS*<br>*Preguntar sobre regulaciones específicas que requieren desarrollarse.*
Snowball – only where research leads recommend it.	Is there anyone else you recommend we should speak to for this research?<br>¿Conoces a alguien más que puedas recomendarme que pueda enriquecer esta investigación?	
Closing the interview	Is there anything else you want to share before we conclude?<br>¿Hay algo más que quisiera compartir antes de concluir?	

	Thank the interviewee, ask them if they have any questions for you, and remind them that we will be transcribing the interview, and they are welcome to view and approve it.<br>Agradecer al entrevistado, preguntar si tienen alguna pregunta y recordarles que la entrevista será transcrita y que ellos están invitados a ver el producto y a aprobarlo si así lo desean	*Make a note if they say they would like to view the interview transcript so that you can remember to get back to them.*<br>*Anotar si el/la entrevistado quisiera ver el transcrito.*

Table S2. Questionnare for Key Stakeholders at a Local Level

Introduction: I am Analía Saker. As I mentioned in the email, this interview is to understand about the sanitation issues in Tumaco and how to tackle them. The interview will take approximately 30 minutes. It is part of my dissertation and aims to understand the regulatory barriers to reach 100% urban sanitation coverage in Colombia. I will try to publish the results in a journal. Your contributions will be anonymised. In the reporting, we would only use either job titles or the institution typology (e.g. urban utility, NGO, municipality of Tumaco, development bank). You are free to withdraw from this research at any time.

Spanish:

Hola, mi nombre es Analía Saker. Como mencioné previamente por email, esta entrevista se realizará para entender los problemas relacionados a saneamiento básico en Tumaco y cómo encararlos. Esta entrevista se demorará aproximadamente 30 minutos y hace parte de un trabajo de investigación de la Universidad de Oxford en Inglaterra. Intentaremos publicar los resultados en una revista académica. Sus contribuciones serán anónimas y el reporte se utilizará únicamente el nombre del cargo o la institución para la que trabaja (por ejemplo: empresa prestadora de servicios públicos domiciliarios, ONG, Alcaldía de Tumaco). Usted es libre de retirarse de esta investigación en cualquier punto.

- Are you happy for us to continue at this time? *Make a note of the consent in your notes.*
  ¿Está de acuerdo con continuar con la entrevista? *Tomar nota de la respuesta*
- We would like to record the interview to enable us to transcribe it. The recording will not be shared, and it will be deleted once transcription is completed and checked. Do you consent to us recording the interview? *Make a note of the consent in your notes and start recording if agreed.*
  Quisiéramos grabar esta entrevista para transcribirla después. La grabación no se compartirá y se borrará una vez la entrevista se haya transcrito. ¿Da su consentimiento para grabar esta entrevista? *Tomar nota de la respuesta*

**Required information**	**Questions**	**Prompts to gather the required information**
Current position details to evidence sampling frame.<br>[it is important in these early questions to build rapport.]	What are your responsibilities in the role your current job?<br><br>¿Cuál es su posición actual?	*What is the job title?*<br>If it is obvious, do not ask this question.<br>*Cuál es el título de dicho trabajo*<br>*What is it that you do in that role? [conversation starter question – note details gathered below]*<br>*Cuáles son sus responsabilidades en ese rol?*
	Are you happy for us to use this job title for reporting purposes?	

	¿Está conforme con que utilice el título de su posición para reportar en la investigación?	
Applicability of CWIS to tackle the urban sanitation issues in Tumaco	What is the sanitation situation in Tumaco city?<br>¿Cuál es la situación de saneamiento básico en la ciudad de Tumaco?<br>How does the city currently deal with wastewater?<br>¿Cuáles son las soluciones actualmente utilizadas por la comunidad para lidiar con sus aguas residuales?	*Ask in terms of coverage and number of people. Ask if they have data in official reports.*<br>*Preguntar en términos de cobertura y averiguar sin tienen información en reportes oficiales que pueda ser compartida*
	What has been the history of the sanitation plans in Tumaco?<br>¿Cuál ha sido la historia de los planes de saneamiento/alcantarillado en Tumaco?	*Ask about specific dates and plans. Designs that have not worked.*<br>*Preguntar sobre fechas específicas y planes, diseños que no hayan funcionado*<br>*Ask what the situation is now. Where are they standing today? What are the plans for the future?*<br>*¿Cuál es la situación ahora? ¿Qué planes hay para el futuro?*
	Do you think the conventional approach is appropriate to tackle urban sanitation in Tumaco?<br>¿Piensa que un enfoque convencional de alcantarillado centralizado es apropiado para solucionar la crisis de saneamiento que actualmente enfrenta la ciudad?	*If so, why, and what do you think would be a better approach.*<br>*Si no, ¿Cuál piensa que podría ser un enfoque más apropiado?*<br>*Have you heard about CWIS?*<br>*¿Ha escuchado de CWIS anteriormente?*
	Are you familiar with the sanitation pilot being implemented in Tumaco?<br>¿Conoce el proyecto piloto que se está implementando en Tumaco en saneamiento básico?	*If yes, What do you know about this project? Have you been involved in the planning phase?*<br>*Si sí, ¿Qué sabe de este proyecto? ¿Ha estado involucrado en el proceso de implementación?*
Barriers to reach 100% urban sanitation coverage in Tumaco?	What do you think the principal barriers that have prevented Tumaco from having an adequate sanitation service?<br>*¿Cuáles piensa que han sido las barreras que han llevado a que Tumaco no tenga un servicio adecuado de saneamiento básico?*	*Do you think it might be a lack of finance, incentives, regulatory barriers, unprepared institutions?*<br>*¿Cree usted que puede ser una falta de financiación, falta de incentivos, barreras regulatorias o instituciones débiles?*
	Do you think the existing regulatory framework poses barriers to reach 100% urban sanitation?<br>¿Piensa que el marco regulatorio actual impide que la ciudad de Tumaco implemente proyectos de saneamiento más acordes a la realidad del territorio?	*If so, how?*<br>*Si sí, ¿De qué manera?*
	In your opinion, what specific regulations should be adapted to incorporate CWIS as a more holistic sanitation approach?<br><br>En su opinión, ¿Qué regulaciones específicas deberían adaptarse para incorporar CWIS como una estrategia más	*Try to make the interviewee refer to specific regulations that are barriers or require further development*<br>*Preguntar sobre regulaciones específicas que requieren desarrollarse.*

	holística para lograr la meta de saneamiento urbano?	
Snowball – only where research leads recommend it.	Is there anyone else you recommend we should speak to for this research?<br>¿Conoces a alguien más que puedas recomendarme que pueda aportar a esta investigación?	
Closing the interview	Is there anything else you want to share before we conclude?<br>¿Hay algo más que quisiera compartir antes de concluir?	
	Thank the interviewee, ask them if they have any questions for you, and remind them that we will be transcribing the interview, and they are welcome to view and approve it.<br>Agradecer al entrevistado, preguntar si tienen alguna pregunta y recordarles que la entrevista será transcrita y que ellos están invitados a ver el producto y a aprobarlo si así lo desean	*Make a note if they say they would like to view the interview transcript so that you can remember to get back to them.*<br><br>*Anotar si el/la entrevistado quisiera ver el transcrito.*

Table S3.Nvivo Node Structure to Analyse Data at a National Level

**Principal Nodes**	**Sub-nodes**
**Is CWIS necessary for Colombia?**	
**Barriers in Regulations**	• Financial<br>• Environmental<br>• Technical<br>• Land Management<br>• Service Delivery<br>• General
**Regulatory Development**	• Financial<br>• Environmental<br>• Technical<br>• Land Management<br>• Service Delivery<br>• General<br>• Monitoring
**No Barriers**	

Table S4. Nvivo Node Structure to Analyse Data at a Local Level

**Principal Nodes**	**Sub-nodes**
**PESTLE analysis**	• Political

	• Economical<br>• Social<br>• Legal<br>• Technical<br>• Environmental
**Possible Solutions**	• Technical<br>• Social<br>• Service Delivery

Supplementary S2. Colombian Sanitation Evaluation

A temporal analysis of the evolution of urban sanitation coverage was conducted to determine if the CWIS approach is appropriate to Colombia. For Colombia, the sanitation coverage data varies depending on the source of information: census, GEIH, or JMP. For the census, the sanitation coverage number is the sum of the households connected to a sewerage network plus the houses that claim to have a septic tank. This information is collected every 10 to 12 years and is the most reliable source of information. In 2018 the census started to consider a septic tank as a sanitation connection, hence the coverage did not include septic tanks in the previous censuses. Furthermore, the government's sanitation coverage numbers, do not consider whether the wastewater and sludge are adequately treated after collection. So far, the type of facility or its treatment is not something the government is reporting or using for decision making, as it is now concerned with increasing coverage. On the other hand, the JMP evaluates access in the different sanitation levels consistent with the SDGs goals: safely managed, basic, limited, unimproved, open defecation. It is helpful for comparing countries as it standardises the information from the different information systems countries have. Nevertheless, it only provides information at a national level, opposite to de census which goes to the municipal level. Finally, the Gran Encuesta Integrada de Hogares (GEIH) is a yearly survey that also collects sanitation information. Yet, it is based on samples and only provides national information. Three sources of information were used as each of them gives different information.

Initially, taking the information gathered by the JMP, a comparison was made with seven Latin American countries. As seen in Figure S1, in the past 20 years, Colombia has strongly improved in basic sanitation coverage, from 80% to 92% in urban contexts, and almost eliminating open defecation. Nonetheless, it has seldom progressed in safely managed sanitation, and it is currently the country with the lowest coverage among the compared nations.

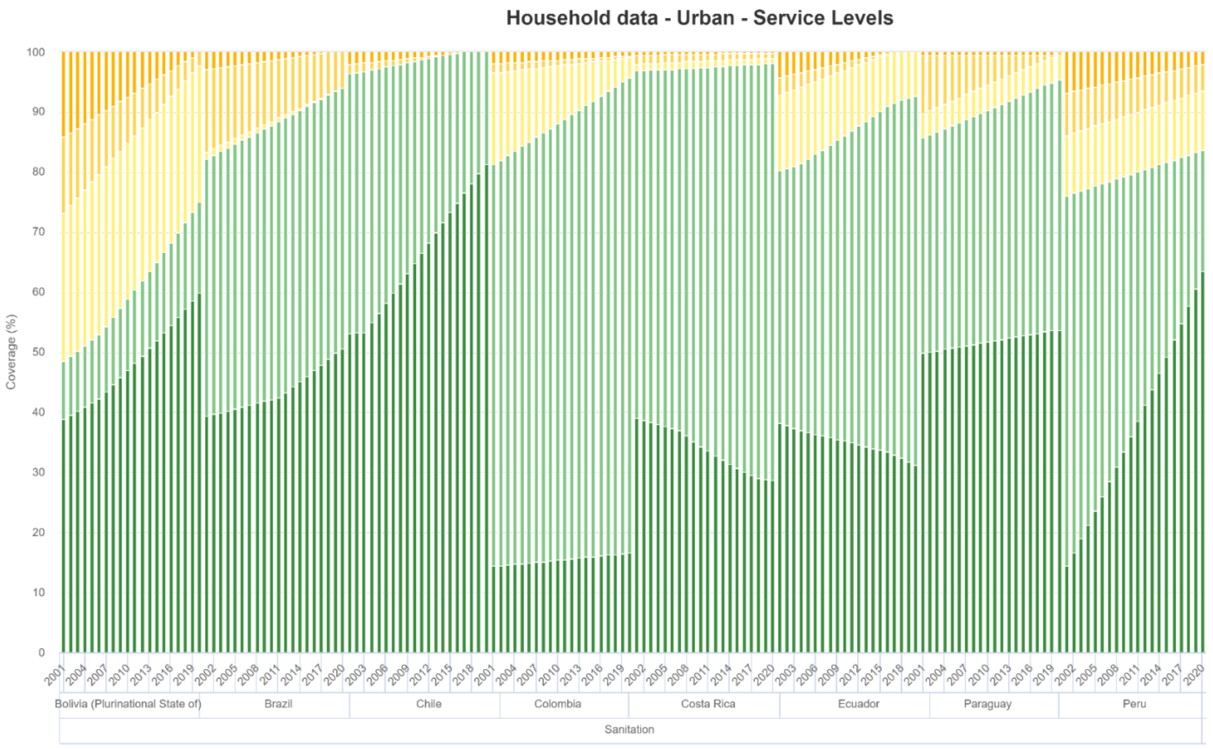

Figure S1. Urban sanitation coverage for eight South American countries. Source: JMP.

Figure S2a shows, according to the census, how sanitation coverage has reached a plateau, where in the last 20 years the progress rate has decreased compared to the 90s decade. Moreover, the GEIH information shows that with the current progress rate and the trend that the sector has had the last 12 years (Figure S2b), by 2030, the sanitation coverage, without considering the quality, sustainability, and treatment of the excreta, will be close to 94%, failing to reach sanitation SDG 6.2 and 6.3. Furthermore, Figure S2c shows how there has not been any significant change in safely managed sanitation coverage and that it has remained stable since 2000.

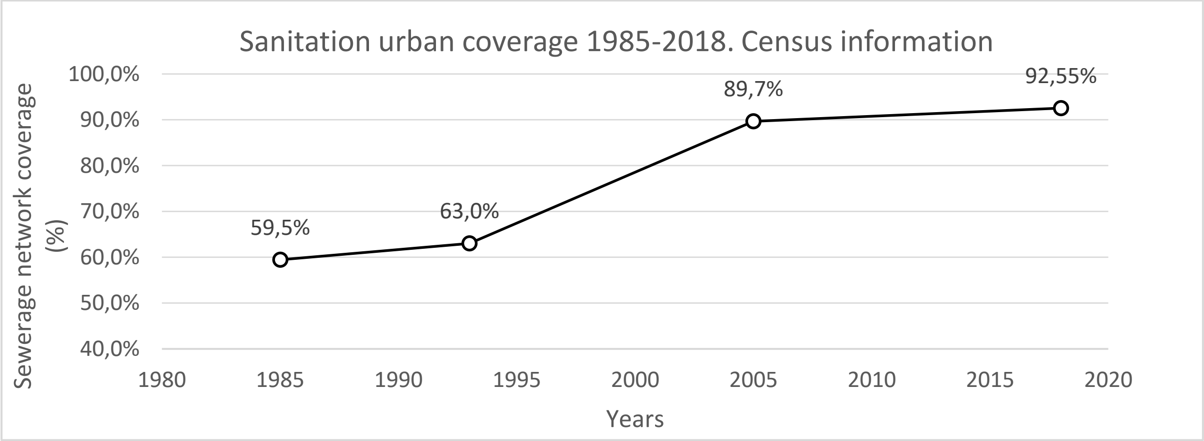

(a)

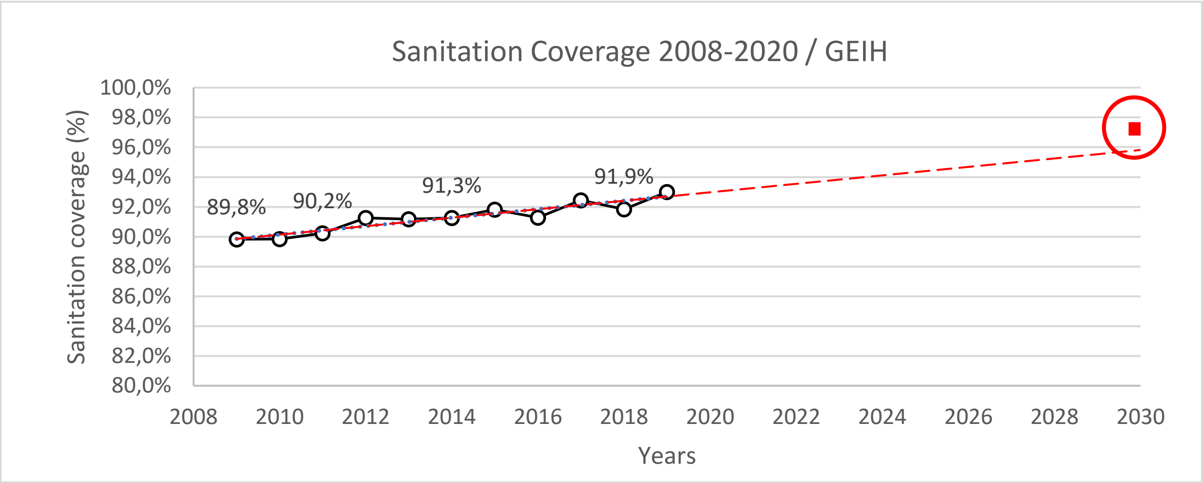

(b)

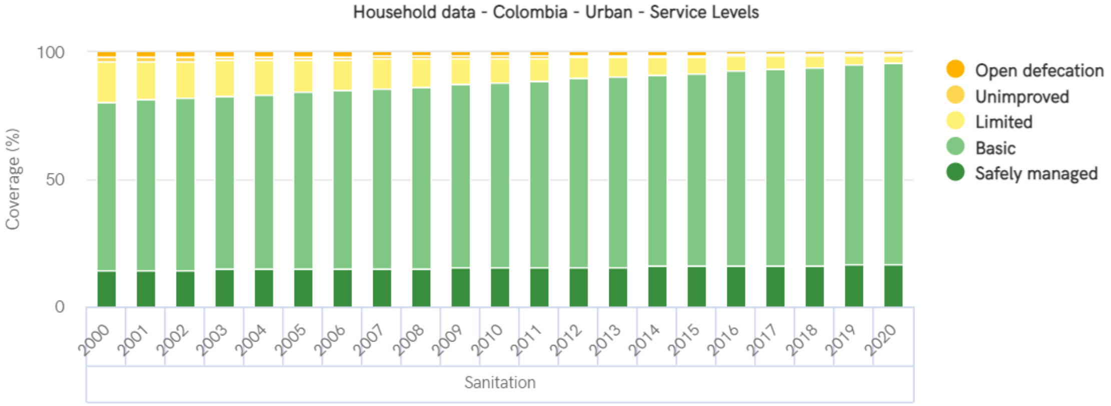

(c)

Figure S2. Evolution of sanitation coverage in Colombia. a. Census b. GEIH c. JMP

Supplementary S3. Tumaco's PESTLE Analysis

Table S5. Tumaco's PESTLE analysis.

**Political**	• Corruption at the municipal level has interrupted the continuity of the mayors, as they usually do not end their period due to sanctions, disrupting the governmental programs<br>• Low confidence in the local government, there are many political groups that usually do not reach consensus and hold control of different institutions that affect water and sanitation service delivery<br>• Planning is deficient, the municipality does not have a clear vision or goals, so investments are usually uncoordinated among governmental entities and resources are wasted<br>• Rivalry between the mayor and the manager of the public utility company generated that the mayor would not send the necessary funds for the utility to work. Mayor retains the subsidy funds that are crucial for the public utility. As long as the rivalry continues, the municipality does not have any interest in strengthening the public utility company<br>• Political will is required at every level to solve the city's sanitation issue: national, departmental and municipal
**Economical**	• The municipality does not make the effort of collecting municipal taxes, making them utterly reliant on limited and contested national funds<br>• Systems will necessarily be costly to manage, considering the conditions of the territory<br>• Royalties scheme changed in 2015, and municipalities do not receive as much money as they received before, limiting their capacity to invest in sanitation<br>• Water provision was prioritised over sanitation, as sanitation was more expensive<br>• Low willingness to pay. At the moment, the fare collection is meagre. The community barely pays for water, so it is not expected that they will pay for sanitation<br>• Public utility is currently facing bankruptcy

**Social**	• Women do not want a shared or communitarian solution as they are afraid of being harassed, and the toilets are not usually well maintained<br>• No one is responsible for shared solutions<br>• Disordered urbanisation, highly populated and dense city<br>• 30% of the people live in stilt houses. They prefer living on the sea, which is important for them as many are fishers and are close to their economic activity. They do not have the incentive to move to a safer zone.<br>• Communities do not value sanitation and do not see it as an essential service to pay for it what it costs.<br>• Lack of trust in institutions at a national and local level<br>• Communities are used to free services as they are generally subsidised in their entirety. Hence, payment culture is inexistent<br>• Several security incidents reported, as there are invisible frontiers and different criminal groups fight over the control of drug routes. Contractors have reported threats that have forced them to abandon projects
**Legal**	• Many legal barriers are being ignored. Some of the planned projects currently do not have a legal basis, putting them in danger of being stopped by the control entities<br>• Environmental regulations are too strict for a pilot, forcing the project to propose more expensive and technically complicated infrastructure.<br>• Many easements' processes are required by law, making the project unviable if they abide by the current regulations
**Technical**	• High operating costs due to the complexity of the technological solution<br>• Many pumping sites, due to the plain topography of the territory<br>• A separate sewer system is required due to the high precipitation in the area.<br>• The conventional solution does not work for the minor networks<br>• It is challenging to implement a sewerage network in the areas of stilt houses.
**Environmental**	• Flat topography.<br>• Water tables are high as the city is partly located over the sea.<br>• Warm weather is one of the zones with the highest precipitation rate in Colombia and the world.<br>• Soil with a low load-bearing capacity, challenging to implement any big infrastructure without robust and costly foundations.<br>• Difficult to get permits from the environmental corporation<br>• Tertiary treatment is required in the WWTP to discharge on the river, as that section of the sea behaves as a lagoon and contaminants are not dispersed to the rest of the sea.<br>• Virtually all wastewater is discharged without any treatment to the sea where people swim and fish.<br>• A considerable portion of the city is under high tsunami vulnerability.
